# Rational Design of α-Fe_2_O_3_ Nanostructures via Single/Dual Polymer-Assisted Hydrothermal Routes for High-Performance Asymmetric Supercapacitors

**DOI:** 10.3390/nano15231774

**Published:** 2025-11-26

**Authors:** Rutuja U. Amate, Aditya A. Patil, Pritam J. Morankar, Chan-Wook Jeon

**Affiliations:** School of Chemical Engineering, Yeungnam University, 280 Daehak-ro, Gyeongsan 712-749, Republic of Korea; rutu.nanoworld@gmail.com (R.U.A.); aditya.nanotechnology@gmail.com (A.A.P.)

**Keywords:** Fe_2_O_3_ nanograins, hybrid surfactant engineering, PVP/PEG-assisted, asymmetric supercapacitor, charge transfer kinetics

## Abstract

In this study, a systematic investigation was undertaken to elucidate the influence of polymeric surfactants such as polyvinylpyrrolidone (PVP), polyethylene glycol (PEG), and their hybrid combination (PVP/PEG) on the structural, morphological, and electrochemical evolution of Fe_2_O_3_ electrodes designed for high-performance supercapacitor applications. Fe_2_O_3_ nanostructures were synthesized via a controlled hydrothermal route, wherein the surfactant composition was precisely tuned to modulate crystal growth, particle dispersion, and surface active-site density. Detailed physicochemical characterization revealed that hybrid PVP/PEG incorporation induced a hierarchically nanograined morphology with optimized porosity. The optimized PVP/PEG-Fe electrode exhibited the largest CV area, lowest equivalent series resistance (0.33 Ω), and superior areal capacitance of 9.17 F cm^−2^ at 8 mA cm^−2^, attributed to accelerated redox kinetics and efficient ion diffusion. Long-term cycling demonstrated remarkable structural resilience, with ~85.1% capacitance retention after 12,000 cycles. Furthermore, an asymmetric pouch-type supercapacitor (PVP/PEG-Fe//AC) was assembled to validate practical performance, achieving a wide potential window of 1.5 V, an areal capacitance of 0.260 F cm^−2^, energy density of 0.081 mWh cm^−2^, and coulombic efficiency of 95.73% after 7000 cycles. This work highlights the critical role of cooperative polymer–metal oxide interactions in achieving structural uniformity, optimized electrochemical kinetics, and long-term durability, offering a versatile strategy for engineering cost-effective, high-performance transition metal oxide electrodes for next-generation flexible energy storage devices.

## 1. Introduction

In a world steadily advancing toward electric mobility, ubiquitous smart electronics, and interconnected energy networks, expectations from energy-storage systems have evolved far beyond conventional charge retention [1,2]. Modern applications demand rapid power delivery, sufficient energy density, long service life, environmental safety, and economic practicality [3,4]. Meeting these diverse requirements through rational material design has therefore become one of the central challenges in current energy-storage research. Within this context, asymmetric supercapacitors (ASCs) are gaining increasing attention as promising candidates [5,6]. They uniquely bridge the gap between batteries and conventional capacitors by offering high power capability while progressively achieving energy densities suitable for practical use. The successful advancement of ASCs depends strongly on the development of electrode materials with well-regulated microstructures, efficient ion and electron transport pathways, and robust mechanical and electrochemical stability over prolonged cycling [7,8].

Among various transition-metal oxides, hematite iron oxide (α-Fe_2_O_3_) has gained significant attention due to its natural abundance, environmental compatibility, and intrinsic Fe^3+^ to Fe^2+^ redox activity. However, the electrochemical performance of pristine α-Fe_2_O_3_ is limited by its low electronic conductivity, slow reaction kinetics, and structural degradation that appears during repeated charge and discharge processes [9,10]. These limitations commonly arise from particle agglomeration, limited accessible active sites, and restricted electrolyte penetration, all of which hinder efficient redox activity and promote mechanical stress during cycling [11,12]. Therefore, to fully harness the electrochemical potential of α-Fe_2_O_3_, careful control over its morphology and nanoscale interfaces is essential. Engineering interconnected nanostructures with open ion-transport channels, well-distributed active sites, and structural flexibility can significantly enhance their charge-storage capability and sustain their integrity during long-term electrochemical operation [13,14,15].

Several prior reports substantiate this concept; for example, Upadhyay et al. fabricated crystalline hydrothermal α-Fe_2_O_3_ nanoparticles with enhanced surface exposure, demonstrating 406 F g^−1^ (CV) and 206 F g^−1^ (GCD), driven by efficient pseudocapacitance and rapid ion motion [16]. Sudarshana et al. transformed Fe_2_O_3_ into an amorphous Fe–O/B hybrid, attaining 1900 F g^−1^ at 1 A g^−1^ with 74.2% retention after 4000 cycles due to abundant defect-rich active sites and Fe/B synergy [17]. Singh et al. engineered α-Fe_2_O_3_ nanocubes delivering 908 F g^−1^ at 2 A g^−1^, while their α-Fe_2_O//NiO device achieved 25.31 Wh k g^−1^ energy density and 70% capacity retention after 1000 cycles, emphasizing morphology-regulated ion transport [18]. Che et al. further advanced Fe_2_O_3_-based dual electrodes, achieving 75.6 Wh kg^−1^, 850.5 W kg^−1^, and outstanding 90.5% cycling retention over 10,000 cycles, demonstrating real-world device feasibility and fast redox kinetics [19]. Together, these findings highlight that controlled structural evolution directly governs Fe_2_O_3_ charge-storage behavior. Despite these significant advances, achieving a balance between high active-site exposure, efficient charge-transport pathways, and mechanical integrity remains a persistent challenge for Fe_2_O_3_-based systems [20]. These requirements cannot be met by surface treatments or post-synthetic modifications alone; instead, they call for synthesis routes that can shape precursor interactions, nucleation events, and nanoscale assembly directly during material formation. In this regard, polymer-assisted hydrothermal synthesis offers a particularly effective pathway. Polyvinylpyrrolidone (PVP) has the ability to coordinate with iron ions, providing spatial control during nucleation and promoting the development of well-defined architectures with suppressed particle aggregation [21]. Polyethylene glycol (PEG), owing to its flexible molecular chains, facilitates interconnected particle growth, enhances electrolyte penetration, and improves interface continuity. When used together, these two polymers do not simply combine their individual effects but rather work cooperatively [22,23]. Their simultaneous presence can regulate nucleation density, guide directional growth, and promote the formation of interconnected and electronically accessible networks with abundant active sites and efficient ion diffusion channels. This synergistic molecular environment is particularly valuable for constructing α-Fe_2_O_3_ frameworks capable of sustaining repeated redox transitions without structural degradation.

Building on this understanding, the present study focuses on the controlled synthesis of α-Fe_2_O_3_ using three engineered polymer-guided pathways: one prepared in the presence of PVP, another with PEG, and a third incorporating both polymers simultaneously to exploit their cooperative influence. By adjusting the polymer environment during hydrothermal growth, we aim to regulate nucleation behavior, crystal evolution, and particle connectivity, ultimately producing α-Fe_2_O_3_ structures with tailored surface characteristics and optimized electroactive sites. The resulting materials are assembled into asymmetric supercapacitor configurations and evaluated to determine how these polymer-directed architectures impact charge-storage behavior, ion-transport dynamics, cycling stability, and overall energy–power characteristics. Alongside demonstrating the performance benefits, this work also offers deeper insight into the molecular-level role of polymers in steering α-Fe_2_O_3_ nanostructure formation, presenting a simple, scalable, and environmentally conscious route for developing advanced iron-oxide electrodes suitable for next-generation, high-efficiency supercapacitors.

## 2. Experimental Section

### 2.1. Materials

Iron chloride hexahydrate (FeCl_3_·6H_2_O), urea (CO(NH_2_)_2_), sodium bromide (NaBr), PVP (Mw ≈ 40,000), and PEG (Mw ≈ 6000) were purchased from Sigma-Aldrich (MO, USA) and used as received. Deionized (DI) water and ethanol were used as solvents. All chemicals were of analytical grade.

### 2.2. Synthesis of Polymer-Assisted α-Fe_2_O_3_ Nanostructures

**Figure 1:**
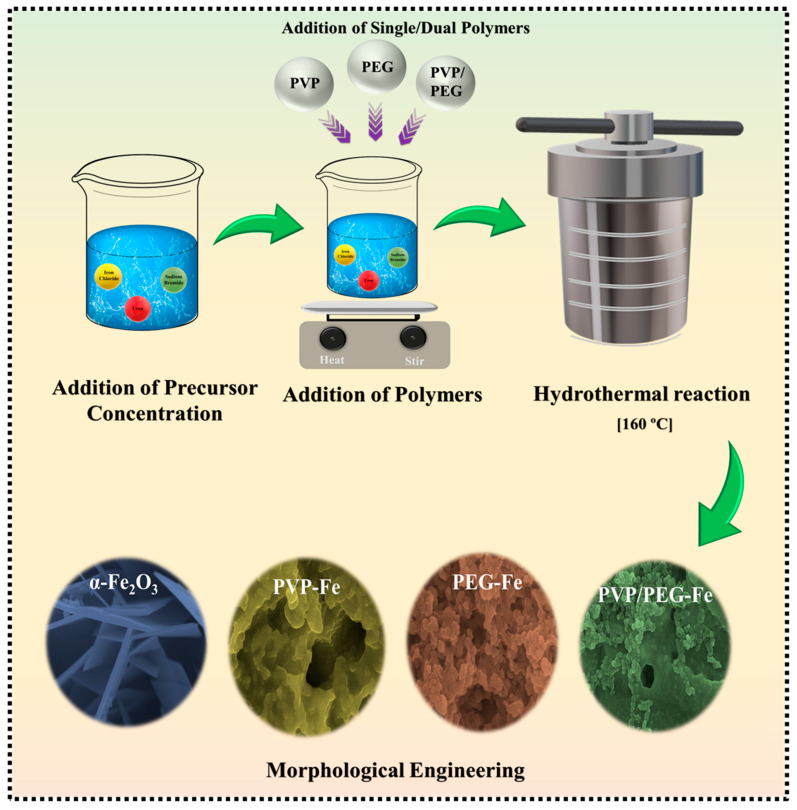
Schematic illustration of the polymer-assisted hydrothermal process for synthesizing α-Fe_2_O_3_ electrodes.

α-Fe_2_O_3_ nanostructures were synthesized via a hydrothermal route. Typically, 0.010 mol of FeCl_3_·6H_2_O, 0.060 mol of (CO(NH_2_)_2_) and 0.020 mol of NaBr were dissolved in 60 mL of DI water under continuous magnetic stirring for 30 min to obtain a clear and homogeneous solution. Depending on the synthesis condition, PVP, PEG, or a mixture of PVP and PEG was added to the precursor solution in a 1:1 molar ratio with Fe^3+^ ions and stirred further for 1 h to ensure complete polymer interaction and uniform complexation with Fe^3+^ species. The well-dispersed precursor solutions were then transferred into 100 mL Teflon-lined stainless-steel autoclaves and maintained at 160 °C for 12 h. After natural cooling to room temperature, the obtained precipitates were separated by centrifugation, washed several times with DI water and ethanol to remove residual ions and unreacted components, and dried at 80 °C. The dried powders were subsequently annealed in air at 300 °C for 2 h to obtain crystalline Fe_2_O_3_. The synthesized samples were designated as bare α-Fe_2_O_3_ (without polymer), PVP–Fe, PEG–Fe, and PVP/PEG–Fe, corresponding to the respective polymer environments. These materials will be further utilized in the subsequent electrochemical study. Figure 1 schematically illustrates the synthesis of α-Fe_2_O_3_ nanostructures using three engineered polymer-assisted pathways.

For electrode fabrication, the as-synthesized α-Fe_2_O_3_ (without polymer), PVP–Fe, PEG–Fe, and PVP/PEG–Fe powders were mixed with acetylene carbon (conductive carbon) and polyvinylidene fluoride (PVDF) binder in a weight ratio of 80:10:10, respectively. N-methyl-2-pyrrolidone (NMP) was used as the solvent to prepare a uniform slurry. Prior to coating, nickel foam substrates (1 cm^2^) were ultrasonically cleaned in acetone, ethanol, and DI water for 10 min each, followed by drying at 60 °C. The prepared slurry was then uniformly applied onto the Ni-foam to achieve a mass loading of ~2 mg cm^−2^ of active material. The coated electrodes were dried at 80 °C for 12 h to ensure complete solvent evaporation and proper adhesion.

## 3. Sample Characterization and Electrochemical Measurements

The structural, morphological, and surface characteristics of the synthesized α-Fe_2_O_3_samples (bare α-Fe_2_O_3_, PVP–Fe, PEG–Fe, and PVP/PEG–Fe) were systematically examined using advanced characterization techniques. X-ray diffraction (XRD, PANalytical diffractometer, Birmimgham, UK Cu Kα radiation, λ = 1.5406 Å) was employed to identify the crystalline phases, assess purity, and evaluate crystallinity. The surface morphology and microstructural features were analyzed using field-emission scanning electron microscopy (FE-SEM, HITACHI S-4800, Tokyo, Japan), while elemental composition and spatial distribution were confirmed through energy-dispersive X-ray spectroscopy (EDS). To avoid surface charging during imaging, a thin platinum coating was applied to the samples. Raman spectroscopy (XploRA Plus, HORIBA Jobin Yvon, France) was utilized to investigate the vibrational modes and structural integrity, whereas X-ray photoelectron spectroscopy (XPS, K-Alpha, Thermo Scientific, Eastleigh, UK) provided insights into the surface composition and oxidation states of the constituent elements. Electrochemical performance was evaluated using a Biologic WBCS3000 (Gières, France) workstation in a three-electrode configuration, with the Fe_2_O_3_-based material as the working electrode, a platinum foil as the counter electrode, and Ag/AgCl as the reference electrode in 2 M KOH aqueous electrolyte. Cyclic voltammetry (CV), galvanostatic charge–discharge (GCD), and electrochemical impedance spectroscopy (EIS, 0.01 Hz–100 kHz) were employed to analyze specific capacitance, rate capability, and charge-transfer resistance. Long-term cycling stability was assessed through extended GCD measurements. For the fabrication of an asymmetric supercapacitor (ASC) device, the optimized PVP/PEG–Fe electrode was employed as the positive electrode, while activated carbon (AC) was used as the negative electrode. The assembled device was systematically evaluated for its overall electrochemical performance, including capacitance, energy density, power density, and long-term cycling stability.

## 4. Results and Discussions

### 4.1. XRD Elucidation

**Figure 2:**
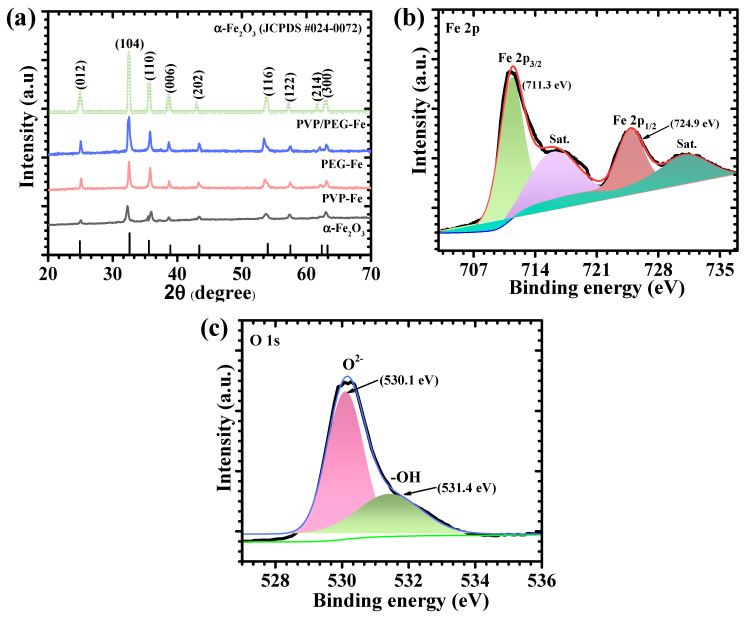
(**a**) XRD pattern of pristine α-Fe_2_O_3_, PVP-Fe, PEG-Fe, PVP/PEG-Fe electrodes. High-resolution spectra of (**b**) Fe2p and (**c**) O1s spectra of PVP/PEG-Fe electrode.

The XRD patterns of the synthesized bare α-Fe_2_O, PVP-Fe, PEG-Fe, and PVP/PEG-Fe samples are shown in Figure 2a. All four samples display a consistent set of diffraction peaks at 24.97° (012), 32.48° (104), 35.72° (110), 38.66° (006), 43.07° (202), 53.93° (116), 57.25° (122), 61.67° (214), and 63.08° (300). These reflections are characteristic of the α-Fe_2_O_3_ (hematite) phase and match well with JCPDS No. 24-0072, confirming that the hydrothermal process yields phase-pure α-Fe_2_O_3_ under all reaction environments. No additional peaks attributable to other iron oxide phases are detected, indicating that the introduction of PVP, PEG, or their combination does not modify the inherent phase formation pathway of α-Fe_2_O_3_ [24]. Although all samples crystallize into the same hematite phase, the influence of the polymers on the crystallization behavior is clearly reflected in the peak shapes and intensities. The PVP/PEG-Fe sample exhibits the sharpest and most intense reflections, suggesting that the simultaneous presence of both polymers facilitates the formation of more crystalline and well-ordered α-Fe_2_O_3_ domains. This enhancement can be related to the complementary effects of PVP and PEG during the hydrothermal process, where coordinated interactions with Fe^3+^ species and steric stabilization promote more uniform nucleation and growth. The PVP-Fe and PEG-Fe patterns also confirm the formation of crystalline α-Fe_2_O_3_, but with slightly broader features, indicating intermediate crystallite sizes or minor structural disorder derived from their individual polymer environments. In contrast, the bare α-Fe_2_O_3_ sample displays broader and weaker peaks, reflecting less controlled crystallization in the absence of polymeric interaction. These observations indicate that the polymer-assisted synthesis conditions, particularly the PVP/PEG-Fe system, have a clear effect on the structural features of α-Fe_2_O_3_ obtained after hydrothermal treatment. The differences observed in the diffraction patterns highlight the role of the polymer environment in influencing nucleation and growth behavior during phase formation [25].

### 4.2. XPS Analysis

X-ray photoelectron spectroscopy was carried out to investigate the surface chemistry of the optimized PVP/PEG-Fe sample, and the corresponding spectra are presented in Figure 2. The Fe 2p spectrum (Figure 2b) exhibits two dominant peaks at approximately 710–711 eV and 723–724 eV, corresponding to Fe 2p_3/2_ and Fe 2p_1/2_, respectively. These values confirm the presence of Fe^3+^, consistent with α-Fe_2_O_3_. A satellite feature is observed around 718–719 eV, further supporting the trivalent oxidation state. No additional peaks attributable to Fe^2+^ or mixed-valence states are detected, indicating uniform iron oxidation throughout the hydrothermally synthesized material. The deconvolution of the Fe 2p_3/2_ peak into multiple components reflects contributions from the lattice environment and minor surface interactions, suggesting a well-defined α-Fe_2_O_3_ structure with minimal defects [26]. The O 1s spectrum (Figure 2c) shows a primary peak at ~529.7–530.1 eV, which is attributed to lattice oxygen (O^2−^) within the Fe–O network. A secondary peak is observed at ~531–532 eV, associated with surface hydroxyl groups or adsorbed oxygen species. The relative intensities indicate a predominance of stable lattice oxygen while maintaining some surface functional groups, which can facilitate electrochemical reactions and enhance electrode wettability. The XPS findings confirm that the PVP/PEG-assisted synthesis method yields a clean and chemically well-defined α-Fe_2_O_3_ surface. The exclusive Fe^3+^ state and stable oxygen environments indicate that the mixed-polymer route fosters uniform surface formation and avoids defect-related variations in chemical state. These features complement the structural results and are consistent with the enhanced electrochemical response exhibited by the PVP/PEG-Fe electrode in subsequent measurements [27,28].

### 4.3. Morphological and Elemental Composition

**Figure 3:**
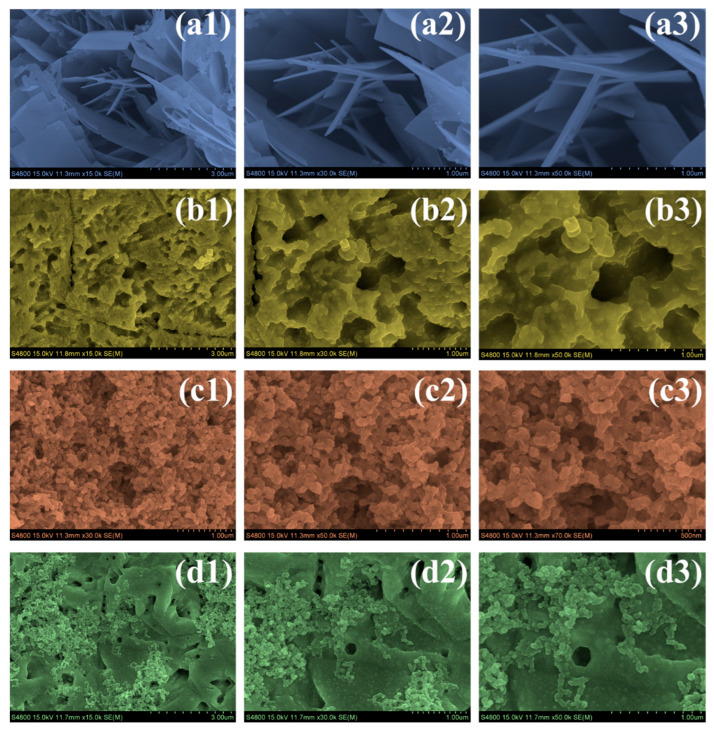
FE-SEM images of (**a1**–**a3**) α-Fe_2_O_3_, (**b1**–**b3**) PVP-Fe, (**c1**–**c3**) PEG-Fe, and (**d1**–**d3**) PVP/PEG-Fe samples at different magnifications.

The FESEM images in Figure 3 illustrate the progressive changes in the surface architecture of α-Fe_2_O_3_ as a function of the polymer environment used during hydrothermal synthesis. The bare α-Fe_2_O_3_ sample (Figure 3(a1–a3)) shows tightly stacked plate-like structures with sharp, rigid edges. Such dense and compact plates indicate unrestricted crystal growth in the absence of polymeric modifiers, resulting in relatively coarse structures with limited internal texturing. Incorporation of PVP leads to a distinct change. The PVP-Fe sample (Figure 3(b1–b3)) develops a much more open framework featuring irregular pores and discontinuous plate fragments. Compared to the compact plates in the bare sample, this morphology reflects the role of PVP in disrupting directional crystal growth, yielding a loosely connected and more porous architecture. A different transformation is observed when PEG is used. The PEG-Fe sample (Figure 3(c1–c3)) consists of compact, densely arranged granular nanoparticles. In contrast to the porous network formed with PVP, PEG favors rapid nucleation and results in a homogeneous distribution of fine grains that merge into an aggregated particulate surface. This shift demonstrates how PEG modulates growth by promoting numerous small nucleation sites rather than extended crystal domains. The synthesis route employing both polymers simultaneously produces a morphology that differs from either polymer alone. As seen in (Figure 3(d1–d3)), the PVP/PEG-Fe sample exhibits an integrated structure in which nanoscale grains are distributed across a moderately porous network. This architecture combines the open features induced by PVP with the more uniform nanoparticle formation characteristic of PEG. The overall structure is balanced, interconnected, and notably more coherent than the individual polymer-derived morphologies. The evolution from the compact plate-like structure of the bare material to the porous framework of PVP-Fe, the densely granular PEG-Fe surface, and finally the mixed porous–nanoparticulate architecture of PVP/PEG-Fe reflects how each polymer environment progressively shapes the growth of α-Fe_2_O_3_. Among these, the morphology obtained with the combined polymers is particularly advantageous for supercapacitor use. The interconnected porous regions allow the electrolyte to penetrate more effectively, while the uniformly distributed nanoscale particles increase the number of accessible surface sites. At the same time, the continuous network created by the dual-polymer route offers pathways that support smoother ion movement through the material. These characteristics provide a structural foundation that enables faster charge transfer and more efficient electrode utilization, which is consistent with the superior electrochemical behavior observed for the PVP/PEG-Fe sample.

**Figure 4:**
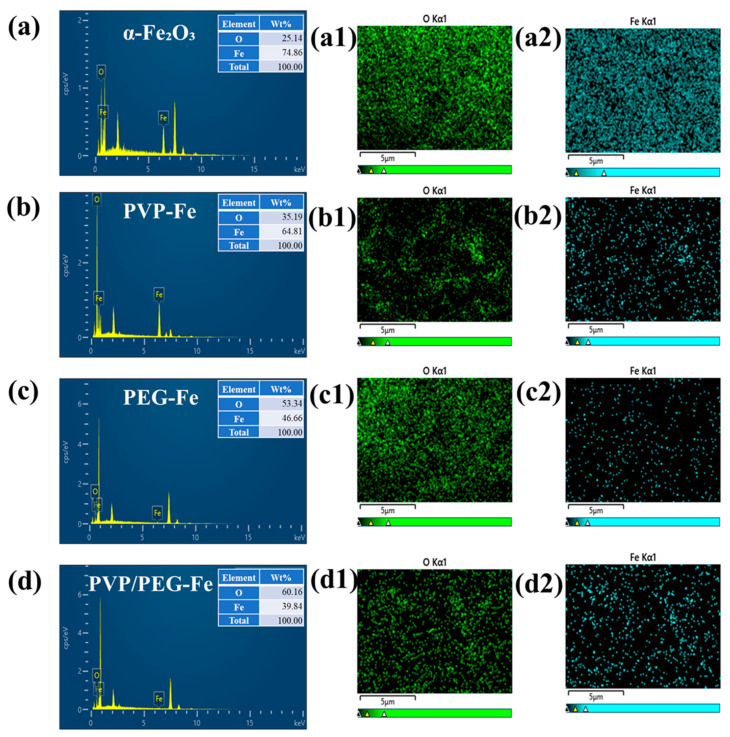
(**a**–**d**) EDS analysis and (**a1**–**d2**) elemental mapping of α-Fe_2_O_3_ samples.

Elemental analysis of the polymer-modified α-Fe_2_O_3_ electrodes, including the pristine sample and variants prepared with PVP, PEG, and dual PVP/PEG, was performed using EDS. The obtained spectra, as shown in Figure 4a–d, distinctly confirm the presence of Fe and O for all samples, validating the successful synthesis of α-Fe_2_O_3_-based electrode materials via the polymer-assisted method. The corresponding insets in each panel present the elemental weight percentages, indicating progressive changes in the O/Fe ratio due to the specific role of each polymer in the hydrothermal process. The relatively lower O/Fe ratio in PVP-Fe may be associated with partial suppression of surface oxidation due to the strong coordination between the carbonyl groups of PVP and Fe^3+^ ions, which can stabilize oxygen-deficient sites. In contrast, the PEG-Fe and PVP/PEG-Fe samples exhibit higher O/Fe ratios, which may arise from enhanced oxygen incorporation promoted by the hydroxyl-rich PEG environment. The hybrid sample shows the highest oxygen content, indicating the synergistic effect of both polymers in modulating surface oxygen chemistry [29,30,31]. To further elucidate the spatial distribution of Fe and O elements, EDS elemental mapping was conducted. The mapping images presented in Figure 4(a1–d2) reveal a homogeneous and uniform dispersion of both O and Fe across the entire electrode surface for all synthesized samples. This uniform elemental allocation demonstrates the efficacy of polymer-mediated hydrothermal synthesis in achieving consistent particle distribution and structural regularity. Such uniformity is a crucial factor in ensuring optimal electrochemical performance, as it minimizes localized defects and facilitates efficient charge transfer throughout the electrode structure.

## 5. Electrochemical Analysis

**Figure 5:**
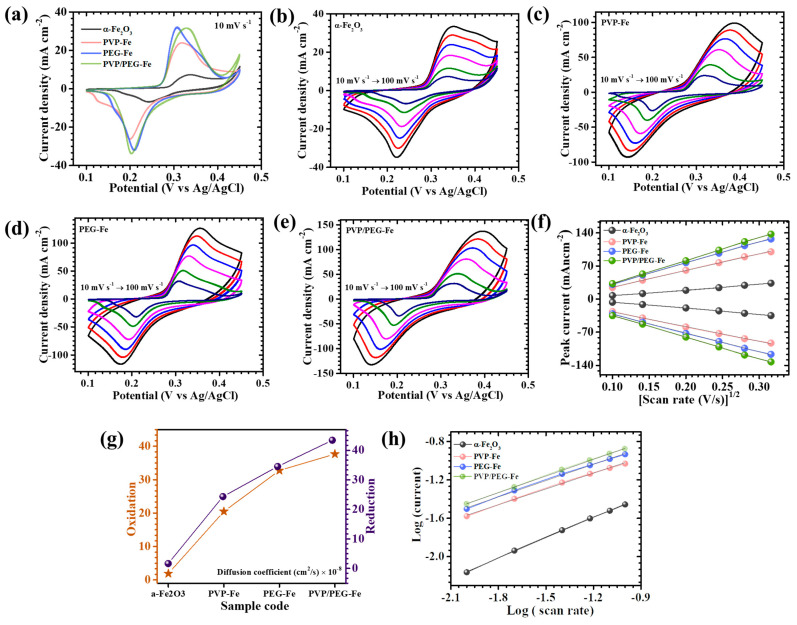
Cyclic voltammetry of (**a**) all α-Fe_2_O_3_ electrodes at a scan rate of 10 mVs^−1^, in a potential window 0.1 to 0.45 V, Cyclic voltammetry of (**b**) α-Fe_2_O_3_, (**c**) PVP-Fe, (**d**) PEG-Fe, (**e**) PVP/PEG-Fe samples at different scan rates (10–100 mVs^−1^), (**f**) Plot of peak current vs. (scan rate)^1/2^, (**g**) Graphical representation of calculated diffusion coefficient, (**h**) Plot of *log*(*i*) against the *log*(*ϑ*).

The electrochemical behavior of α-Fe_2_O_3_ electrodes synthesized in the presence and absence of different polymeric surfactants; PVP, PEG and a hybrid combination of PVP/PEG was systematically investigated to elucidate the effect of polymer-assisted morphology control on supercapacitor performance. CV, GCD, and EIS analyses were carried out in a standard three-electrode configuration using 2 M KOH aqueous electrolyte. Figure 5a presents the CV profiles of all electrodes recorded within a potential window of 0.1 V to 0.45 V at a scan rate of 10 mV s^−1^. All electrodes exhibit distinct and well-defined redox peaks, which clearly indicate the faradaic nature of charge storage associated with reversible redox transitions of iron species (Fe^3+^/Fe^2+^) in α-Fe_2_O_3_. The presence of these redox features confirms that the overall charge storage process is governed by pseudocapacitive behavior rather than pure double-layer capacitance. To gain deeper insight into the rate-dependent response, CV measurements were further performed over a series of scan rates ranging from 10 to 100 mV s^−1^ for bare α-Fe_2_O_3_, PVP-Fe, PEG-Fe, and PVP/PEG-Fe samples, as illustrated in Figure 5b–e. All electrodes exhibit a gradual increase in current density with increasing scan rate while maintaining the characteristic shape of their respective CV profiles. This behavior reflects the excellent reversibility and mechanical integrity of the electrodes, implying stable electrochemical kinetics and rapid redox reactions even under high charge-discharge rates. The nearly symmetric and reproducible nature of the CV curves for all polymer-assisted electrodes further confirms their efficient ion diffusion and favorable charge transfer dynamics within the active material [32]. Among all studied electrodes, the hybrid PVP/PEG-Fe electrode shows the most pronounced redox peak intensity and the largest enclosed CV area, clearly signifying its superior charge storage capability. This remarkable enhancement can be attributed to the cooperative role of PVP and PEG during synthesis, which consistently optimize the surface chemistry, particle interconnectivity, and overall porosity of the α-Fe_2_O_3_ nanostructure. The synergistic interaction between the two polymers promotes homogeneous nucleation and growth, leading to a uniform, open, and densely granular that maximizes electrolyte penetration and utilization of electroactive sites [33]. Conversely, the PVP-Fe and PEG-Fe samples exhibited less favorable morphologies. The PVP-Fe electrode showed a relatively open framework featuring irregular pores and discontinuous plate fragments, which limited electrolyte access and reduced the number of active redox centers. In contrast, the PEG-Fe electrode displayed denser and compactly arranged granular nanoparticles features that impeded efficient ion transport and electrolyte diffusion within the electrode matrix. The bare α-Fe_2_O_3_ electrode, synthesized without any polymeric surfactant, demonstrated a compact and rigid surface morphology, leading to minimal charge storage activity and poor electrochemical reversibility due to restricted active surface exposure. The pseudocapacitive redox process that governs charge storage in α-Fe_2_O_3_-based electrodes can be expressed by the following reversible reaction (1) [34]

(1) $${Fe}_{2}O_{3}+{OH}^{-}⇌{Fe}_{2}O_{3}OH+e^{-}$$

This redox process reflects the participation of Fe^3+^/Fe^2+^ redox couples, where hydroxide ions (OH^−^) are reversibly inserted and extracted from the α-Fe_2_O_3_ lattice during charge and discharge cycles [35]. The continuous of this redox transition determines the electrode’s pseudocapacitive performance, which is strongly influenced by the surface area, porosity, and ion diffusion paths of the material. In this regard, the hybrid PVP/PEG-Fe electrode exhibits an optimized balance between structural integrity and electrochemical accessibility.

A comprehensive investigation into the redox kinetics and ion diffusion dynamics of α-Fe_2_O_3_ electrodes was carried out through CV measurements recorded at a series of varying scan rates. As presented in Figure 5f, a distinct and nearly linear relationship was observed between the anodic and cathodic peak currents (*i_p_*) and the square root of the scan rate (*v*^1/2^) for all electrode samples. This consistent linear dependency clearly indicates that the electrochemical charge storage process in α-Fe_2_O_3_ is predominantly governed by diffusion-controlled behavior, wherein the rate of electron transfer is closely coupled to the diffusion of ions within the electrode-electrolyte interface. To further quantify the ion transport behavior and evaluate the extent of diffusion control, the apparent diffusion coefficients (*D*) of the active species were calculated using the Randles-Sevcik Equation (2) [33]:

(2) $$D^{1/2}=\frac{i_{p}}{2.69\times 10^{5}\times n^{3/2}\times A\times C\times v^{1/2}}$$

In this expression, *n* represents the number of electrons exchanged per redox event, *A* is the electrochemically active surface area of the electrode, *C* denotes the molar concentration of electroactive species in the electrolyte, and *ν* corresponds to the scan rate. The calculated D values obtained at a controlled scan rate of 10 mV s^−1^ are summarized in Table 1, while their comparative trends are graphically illustrated in Figure 5g. A clear enhancement in diffusion kinetics was observed for the PVP/PEG-Fe electrode, which exhibited the highest diffusion coefficient among all compositions. This finding underscores the superior ionic mobility and faster charge transfer kinetics facilitated by the synergistic interaction of PVP and PEG during synthesis. In contrast, electrodes synthesized with single-polymer surfactants (PVP-Fe and Fe-PG) demonstrated comparatively lower diffusion coefficients, reflecting slower ion transport and less efficient electron exchange. The PVP-Fe electrode, in particular, displayed a limited diffusion rate owing to its underdeveloped surface morphology and restricted electroactive area, arising from incomplete surfactant-assisted structural evolution. Similarly, the PEG-Fe electrode exhibited moderately improved behavior but remained inferior to the hybrid PVP/PEG-Fe sample, likely due to partial aggregation and suboptimal porosity that impeded uniform ion diffusion.

To gain a deeper understanding of the intrinsic charge storage behavior of the α-Fe_2_O_3_ electrodes, the dependence of peak current on scan rate was analyzed using the well-established power-law relationships expressed in Equations (3) and (4) [36]:

(3) $$i\;={av}^{b}$$

(4) $$\log\left(i\right)=\log\left(a\right)+b\;log(v)$$

In these equations, *i* represents the measured peak current at a given scan rate *v*, while the parameters *a* and *b* provide insight into the underlying charge storage mechanism. The value of the b-coefficient, in particular, serves as a diagnostic factor to differentiate between diffusion-controlled and capacitive-dominated processes. A b-value close to 0.5 typically signifies a diffusion-controlled faradaic process, whereas a value approaching 1.0 corresponds to surface-limited capacitive behavior [37]. The linear fitting of *log*(*i*) versus *log*(*v*) (Figure 5h) resulted in b-values ranging between 0.54 to 0.70 for all α-Fe_2_O_3_-based electrodes, as summarized in Table 1. These intermediate b-values confirm that charge storage in α-Fe_2_O_3_ electrodes is primarily governed by diffusion-assisted faradaic reactions, while a minor portion of surface-driven capacitive contribution also coexists. This mixed charge storage behavior is commonly observed in transition-metal oxide electrodes, where both surface redox reactions and ion intercalation processes participate in the overall pseudocapacitive response [37].

To unravel the quantitative contributions of surface-controlled and diffusion-limited mechanisms, the total current response was divided using the relation (5) [38]:

(5) $$i\left(V\right)=k_{1}v{+k_{2}v}^{1/2}$$

Here, *k*_1_*v* denotes the capacitive contribution arising predominantly from surface-adsorbed species and electrostatic interactions at the electrode-electrolyte interface, while *k*_2_*v*^1/2^ represents the diffusion-controlled component associated with ion intercalation and faradaic redox reactions within the bulk of the active material. The constants *k*_1_ and *k*_2_ were obtained by plotting *i*(*V*)/*v*^1/2^ versus *v*^1/2^, allowing the separation and quantitative estimation of each charge storage contribution. Based on this analysis, the total charge (*Q_t_*) can be expressed as the sum of surface capacitive charge (*Q_s_*) and diffusion-controlled charge (*Q_d_*), as indicated in Equation (6) [38]:

(6) $$Q_{t}=Q_{s}+Q_{d}$$

**Figure 6:**
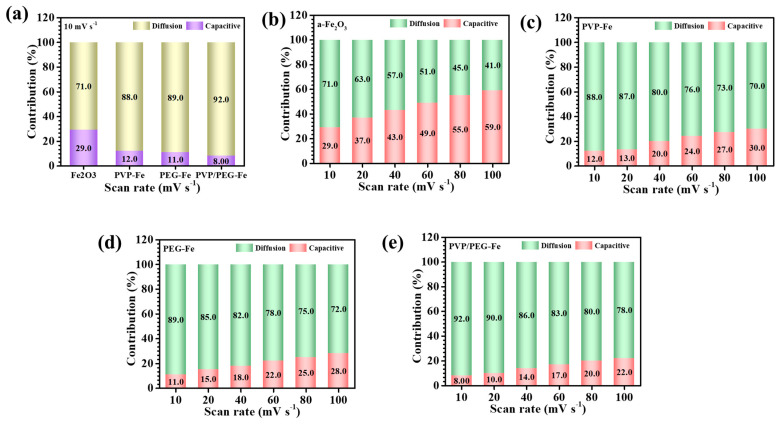
Capacitive and diffusion-controlled processes (**a**) at 10 mVs^−1^ of all α-Fe_2_O_3_ electrodes, at different scan rates of (**b**) α-Fe_2_O_3_, (**c**) PVP-Fe, (**d**) PEG-Fe and (**e**) PVP/PEG-Fe.

The respective charge proportions calculated at a scan rate of 10 mV s^−1^ for the bare α-Fe_2_O_3_, PVP-Fe, PEG-Fe, and PVP/PEG-Fe electrodes were 29.0/71.0%, 12.0/88.0%, 11.0/89.0%, and 8.0/92.0%, for capacitive and diffusion contributions, respectively (Figure 6a). These quantitative results compellingly demonstrate that the incorporation of hybrid PVP/PEG surfactant dramatically enhances the diffusion-governed charge contribution, emphasizing the dominance of faradaic redox reactions in the PVP/PEG-Fe electrode. The exceptionally high diffusion-controlled fraction of approximately 92.0% recorded for PVP/PEG-Fe at 10 mV s^−1^ highlights its superior ionic diffusivity and highly efficient redox kinetics. This behavior is characteristic of transition-metal-oxide pseudocapacitors, where enhanced diffusion contribution typically correlates with superior electrochemical kinetics and higher capacitance performance. This remarkable performance can be attributed to its optimized nanogranular morphology, which forms a well-interconnected and porous framework. Such an architecture provides abundant open channels for electrolyte penetration and ensures intimate contact between the electrolyte ions and electroactive surfaces, thereby minimizing diffusion resistance and facilitating rapid ion transport throughout the electrode. Furthermore, the influence of scan rate on charge storage dynamics was systematically examined in the range of 10–100 mV s^−1^ for all samples (Figure 6b–e). As the scan rate increased, a gradual rise in capacitive contribution was observed for each electrode, reflecting the typical transition from diffusion-dominated to surface-controlled charge storage at higher sweep rates. This shift is attributed to the restricted diffusion depth of electrolyte ions under faster potential variations, which limits ion intercalation into deeper active sites and instead favors rapid surface-level redox reactions [34]. The PVP/PEG-Fe electrode, despite showing a relative increase in surface contribution at higher scan rates, retained a substantially larger fraction of diffusion-driven charge storage compared to the other samples, indicating superior ion accessibility and stable electrochemical reversibility.

**Figure 7:**
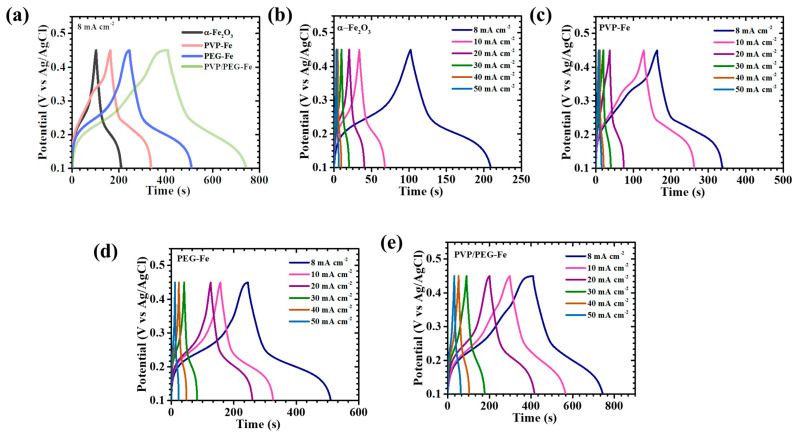
GCD curves of (**a**) α-Fe_2_O_3_ electrodes at 8 mA cm^−2^ current density, GCD plot of (**b**) α-Fe_2_O_3,_ (**c**) PVP-Fe, (**d**) PEG-Fe, and (**e**) PVP/PEG-Fe electrodes at different current densities.

**Figure 8:**
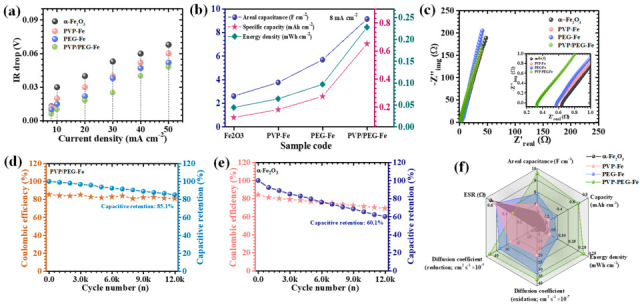
(**a**) IR-drop analysis illustrating internal resistance losses. (**b**) Plot comparing areal capacitance, specific capacity, energy density of α-Fe_2_O_3_ electrodes. (**c**) Nyquist plot of α-Fe_2_O_3_ electrodes, Cyclic stability over 12,000 GCD cycles of (**d**) PVP/PEG-Fe and (**e**) α-Fe_2_O_3_ samples, (**f**) Radar chart of the key electrochemical parameters for pristine PVP/PEG-Fe electrodes.

The GCD behavior of the α-Fe_2_O_3_-based electrodes was further analyzed to evaluate their charge storage efficiency and rate-dependent electrochemical response. Figure 7a presents the comparative GCD profiles recorded at a current density of 8 mA cm^−2^ within the potential range of 0.1 to 0.45 V, clearly defining electrode-specific differences in electrochemical characteristics. Subsequent GCD measurements were extended across a broader current density range of 8 to 50 mA cm^−2^ for pristine α-Fe_2_O_3_ and its polymer-modified counterparts incorporating PVP, PEG, and the hybrid PVP/PEG (Figure 7b–e). All electrodes exhibited nonlinear discharge curves accompanied by distinct voltage plateaus, confirming the predominance of faradaic charge storage governed by diffusion-controlled redox reaction behavior typical of battery-type pseudocapacitive electrodes [39]. Among the studied samples, the PVP/PEG-Fe electrode revealed the most prominent nonlinearity with smooth and gradual potential decay, signifying enhanced pseudocapacitive activity arising from reversible ion intercalation and surface-confined redox transitions [40]. Moreover, the PVP/PEG-Fe electrode displayed a noticeably extended discharge duration compared to the pristine α-Fe_2_O_3_, PVP-Fe, and PEG-Fe electrodes, which directly reflects its superior energy storage capability. Across all electrodes, the nearly symmetric charge-discharge patterns denote high coulombic efficiency and robust electrochemical reversibility, indicative of stable ion diffusion and minimal polarization during cycling. Particularly, the PVP/PEG-Fe electrode exhibited the lowest IR-drop at the onset of the discharge process, accompanied by an almost perfectly symmetric GCD profile. These features confirm its outstanding electrical conductivity, effective charge transfer dynamics, and reduced internal resistance. The IR-drop trends observed across various current densities (Figure 8a) further reinforce this finding: decreasing current densities consistently led to reduced IR-drop values for all electrodes, revealing diminished ohmic losses and improved electrolyte-electrode interfacial contact. Notably, PVP/PEG-Fe consistently demonstrated the smallest IR-drop among all compositions, corroborating its highly optimized charge transport pathways and excellent electrochemical kinetics. To quantify the electrochemical performance with precision, the areal capacitance (*C_A_*), energy density (*ED*), and power density (*PD*) were precisely calculated using integrated formulations tailored for the nonlinear GCD curves (7–9) [41,42]:

(7) $$C_{A}=\frac{I\times 2\times \int V\left(t\right)dt}{A\times {\left(∆V\right)}^{2}}$$

(8) $$ED=\frac{1}{2\times 3600}\;C_{A}\times {dV}^{2}$$

(9) $$PD=\frac{ED\times 3600}{T_{d}}$$

In these relations, *I* is the discharge current, *∫V*(*t*)*dt* represents the integral of the potential over the discharge duration to accurately account for nonlinear charge-discharge profiles, *A* denotes the electrochemically active surface area, and Δ*V* is the applied potential window. This computational approach is particularly crucial for pseudocapacitive materials, where nonlinear discharge characteristics stem from faradaic reactions rather than ideal double-layer behavior. At a current density of 8 mA cm^−2^, the calculated areal capacitances were 2.6 F cm^−2^ for pristine α-Fe_2_O_3_, 3.75 F cm^−2^ for PVP-Fe, 5.68 F cm^−2^ for PEG-Fe, and a notably higher 9.14 F cm^−2^ for PVP/PEG-Fe (Table 2, Figure 8b). In addition to the areal capacitance, the mass-specific capacitance of the electrodes was calculated using the total active mass per unit area (Table 2). The PVP/PEG-Fe electrode exhibited the greatest capacitance among all tested compositions, directly validating the structural and interfacial advantages conferred by the hybrid PVP/PEG surfactant system. Its superior charge storage capability arises from several synergistic factors, including, an enlarged electrochemically active surface area exposing abundant redox sites, enhanced electron mobility through well-connected α-Fe_2_O_3_ stacked rigid plate ensuring low charge-transfer resistance, and improved ion diffusion owing to open, interconnected pore channels that facilitate electrolyte penetration. The dual polymeric role of PVP and PEG is critical in maintaining the integrity of this structure; the hybrid surfactant not only regulates particle nucleation to prevent agglomeration but also inhibits nanosheet restacking, thereby sustaining the porous framework essential for rapid ionic motion and efficient redox activity [43]. As expected, all electrodes exhibited gradual declines in capacitance with increasing current density (Table 2), primarily due to restricted diffusion of electrolyte ions and the limited utilization of deeper electroactive sites under high-rate conditions. Nevertheless, the PVP/PEG-Fe electrode demonstrated exceptional rate capability, retaining approximately 53.57% of its initial capacitance even at 50 mA cm^−2^, a clear indication of its robust structural stability and superior ion transport efficiency.

EIS was performed at an excitation amplitude of 10 mV to elucidate the interfacial charge transfer characteristics and ionic transport behavior of the fabricated electrodes (Figure 8c). The obtained Nyquist plots, representing the relationship between the real (Z′) and imaginary (−Z″) components of impedance, provide critical insight into both resistive and capacitive elements governing electrode kinetics. The high-frequency intercept on the real axis corresponds to the equivalent series resistance (ESR), which reflects the combined effects of the intrinsic electrode resistance, electrolyte resistance, and interfacial contact resistance [44]. As detailed in Table 1, the PVP/PEG-Fe electrode exhibited the lowest ESR value of 0.33 Ω, confirming its superior electrical conductivity and efficient ion diffusion pathways. This remarkable reduction in resistance is a direct consequence of the homogeneous nanograins morphology achieved through PVP/PEG-assisted synthesis. The optimized porous and interconnected architecture effectively minimizes charge transfer barriers, thereby facilitating rapid electron transport and superior electrochemical kinetics.

To further evaluate the electrochemical endurance of the optimized PVP/PEG-Fe electrode, long-term galvanostatic cycling tests were performed for 12,000 consecutive charge–discharge cycles at a current density of 80 mA cm^−2^ (Figure 8d). Impressively, the electrode preserved nearly 85.1% of its initial capacitance, reflecting only a modest 14.09% loss after prolonged operation. Such excellent cycling retention emphasizes the outstanding structural stability, mechanical resilience, and reversible redox behavior of the electrode. To evaluate the enhanced cycling stability demonstrated by the PVP/PEG-assisted design, the performance of the bare α-Fe_2_O_3_ electrode was also considered for comparison. As shown in Figure 8e, the α-Fe_2_O_3_ electrode exhibits a more rapid decline in capacitance over repeated charge-discharge cycles, with a tentative capacitive retention of ~60.1% after 12,000 cycles, whereas the PVP/PEG-Fe electrode retains ~85% under identical conditions. This comparison clearly demonstrates that the hybrid polymer-assisted morphological control and design significantly improve the structural robustness and reversibility of the electrode, thereby enhancing long-term electrochemical performance. The exceptional durability is primarily attributed to the nanogranular framework, precisely engineered through controlled PVP/PEG incorporation during synthesis. This nanogranular architecture accommodates volumetric expansion and contraction during repetitive ion insertion/extraction, thereby suppressing structural degradation and preserving interfacial integrity. Additionally, the open and interconnected channels promote effective electrolyte infiltration and rapid ion diffusion throughout the electrode matrix, further sustaining high electrochemical efficiency during extended cycling. The minor capacitance deteriorating observed after extensive cycling likely arises from gradual ion trapping or accumulation within micro-porous domains and interface sites, which limits the accessibility of electroactive regions and slightly impedes reversible faradaic reactions [45]. Nonetheless, the electrode’s high retention and stable morphology confirm the robustness of the PVP/PEG-assisted design in mitigating stress-induced degradation and maintaining continuous redox activity. Overall, these findings underscore the critical importance of rational structural engineering in enhancing the charge transport efficiency, stability, and longevity of α-Fe_2_O_3_-based pseudocapacitive materials, key attributes for advancing next-generation high-performance supercapacitor devices.

The radar plot illustrated in Figure 8f provides a comprehensive multidimensional comparison of key electrochemical parameters including, areal capacitance, energy density, diffusion coefficient, and ESR, for the bare α-Fe_2_O_3_, PVP-Fe, Fe-PG, and PVP/PEG-Fe electrodes. This graphical representation clearly highlights the superior and well-balanced performance of the PVP/PEG-Fe electrode across all evaluated metrics. The notably expanded and symmetrical profile of PVP/PEG-Fe demonstrates its exceptional integration of high charge storage capacity, efficient ionic transport, and minimal internal resistance, collectively confirming the successful optimization of its nanogranular and porous architecture for enhanced electrochemical functionality. Notably, electrodes synthesized with individual surfactants (either PVP or PEG) exhibited limited performance due to unbalanced morphological control, PVP alone often leading to loosely packed structures with insufficient conductivity, while PEG tends to promote particle aggregation and restricted ion accessibility. In contrast, the hybrid PVP/PEG system synergistically combines the film-forming and stabilizing characteristics of PVP with the pore-directing and dispersing abilities of PEG. This cooperative effect facilitates the formation of a nanogranular and porous network that optimizes both electron transport and electrolyte diffusion pathways, thereby establishing PVP/PEG-Fe as a uniquely engineered electrode with superior electrochemical characteristics.

## 6. Electrochemical Performance of Asymmetric Supercapacitor Device

**Figure 9:**
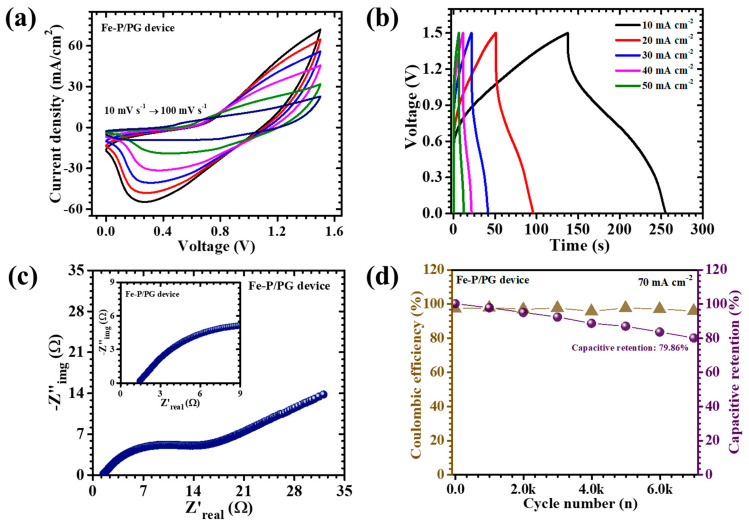
(**a**) CV tests performed on the PVP/PEG-Fe//AC device recorded at a scan rate of 10–100 mVs^−1^ across a potential range of 0 to 1.5 V, (**b**) GCD measurements at different current densities for PVP/PEG-Fe//AC device, (**c**) EIS measurement of the device, (**d**) Cyclic stability of 7000 GCD cycles of PVP/PEG-Fe AC device.

To extend the laboratory-scale electrochemical evaluation toward practical implementation, an asymmetric pouch-type supercapacitor device (APSD) was fabricated using the optimized PVP/PEG-assisted α-Fe_2_O_3_ electrode as the positive terminal and activated carbon (AC) as the negative terminal. The AC electrode was deliberately selected for its reliable electric double-layer capacitance, effectively complementing the faradaic pseudocapacitive behavior of α-Fe_2_O_3_ to establish a balanced hybrid charge storage system. Both electrodes were fabricated on nickel foam current collectors, chosen for their superior electrical conductivity and mechanical integrity. For the negative electrode preparation, a homogeneous slurry was formulated by blending acetylene black as the conductive carbon additive, polyvinylidene fluoride (PVDF) as a polymeric binder, and N-methyl-2-pyrrolidone (NMP) as the dispersing solvent. The mixture was thoroughly homogenized to ensure even dispersion of all constituents, then uniformly coated onto the NF substrate and subsequently dried at 60 °C for 12 h to achieve complete solvent removal. The assembled pouch cell utilized a 2 M KOH aqueous electrolyte, with filter paper serving as both the separator and electrolyte reservoir. The pouch was hermetically sealed to prevent electrolyte evaporation and atmospheric contamination, ensuring operational stability during long-term testing. Electrochemical characterization of the device was systematically performed using CV, GDC, and EIS. The CV profiles (Figure 9a), recorded across scan rates ranging from 10 to 100 mV s^−1^, exhibited quasi-rectangular shapes interspersed with distinct redox peaks, confirming the coexistence of surface-controlled capacitive behavior from the AC electrode and diffusion-driven pseudocapacitance from Fe_2_O_3_. The device maintained excellent electrochemical stability across an extended potential window of 1.5 V, which is notably broad for aqueous systems. This enhancement originates from the synergistic interplay between the high redox activity of the α-Fe_2_O_3_ electrode and the extensive surface area of the AC counterpart, allowing efficient utilization of both faradaic and non-faradaic charge storage mechanisms.

The corresponding GCD curves (Figure 9b) further corroborated these results, displaying nonlinear charge-discharge profiles characteristic of pseudocapacitive behavior. At a current density of 10 mA cm s^−2^, the APSD achieved an impressive areal capacitance of 0.260 F cm^−2^, accompanied by an energy density of 0.081 mWh cm^−2^ and a power density of 1.24 mW cm^−2^ (Table 3). These values substantiate the efficient energy–power balance achieved through hybrid electrode engineering. EIS analysis (Figure 9c) provided deeper insight into charge transport and interface dynamics. The Nyquist plot exhibited a small semicircular arc in the high-frequency region, indicative of low charge-transfer resistance, followed by a steep linear segment at low frequencies, characteristic of ideal capacitive response. The ESR was found to be only 1.44 Ω, confirming excellent electrical conductivity and rapid ion diffusion within the electrode structure. This low resistance is directly linked to the uniform porous network formed by the synergistic action of PVP and PEG, which collectively enhance both electron mobility and electrolyte accessibility. To evaluate durability, cycling stability tests were conducted at a current density of 70 mA cm^−2^ over 7000 continuous charge-discharge cycles (Figure 9d). Impressively, the device retained 79.86% of its initial capacitance while maintaining a coulombic efficiency of 95.73%, attesting to its remarkable reversibility and structural endurance. The outstanding cycling stability arises from the nanograins and porous framework of the PVP/PEG-engineered α-Fe_2_O_3_ electrode, which effectively accommodates volume fluctuations during redox cycling and preserves interfacial integrity over prolonged operation. Overall, the α-Fe_2_O_3_ (PVP/PEG)//AC asymmetric supercapacitor demonstrates remarkable balance of high capacitance, extended potential window, low internal resistance, and excellent long-term stability, underscoring the transformative impact of hybrid polymeric surfactant engineering. These results position the PVP/PEG-Fe electrode as a promising candidate for next-generation flexible and portable energy storage systems, bridging the gap between laboratory research and practical high-performance supercapacitor technologies.

## 7. Conclusions

This comprehensive study establishes a clear structure–property–performance correlation for Fe_2_O_3_ electrodes modulated by polymeric surfactants, elucidating how surfactant chemistry dictates nanoscale morphology, charge transport, and overall electrochemical efficiency. Among all investigated systems, the hybrid PVP/PEG-assisted Fe_2_O_3_ electrode demonstrated superior physicochemical and electrochemical attributes, owing to the synergistic influence of PVP’s surface-stabilizing capability and PEG’s pore-directing characteristics. This cooperative interaction yielded a highly uniform, porous, and conductive nanograin architecture that facilitated rapid ion diffusion and reversible redox transitions. Electrochemical analyses confirmed diffusion-dominant pseudocapacitive behavior with minimal resistance losses, as evidenced by a low ESR of 0.33 Ω and significantly enhanced diffusion coefficients. The PVP/PEG-Fe electrode further exhibited exceptional cycling stability, maintaining over 85.1% of its initial capacitance after 12,000 charge–discharge cycles, emphasizing the mechanical robustness and interfacial stability imparted by the hybrid surfactant system. The successful assembly of the PVP/PEG-Fe//AC asymmetric device strengthened the electrode’s practical applicability, delivering high energy and power densities (0.081 mWh cm^−2^ and 1.24 mW cm^−2^, respectively) and operating stably within an extended 1.5 V potential window. Overall, the findings not only advance the understanding of polymer-oxide interfacial engineering but also provide a scalable and adaptable strategy for fabricating high-performance, flexible supercapacitor systems capable of meeting the demands of next-generation portable and wearable energy technologies.

## Figures and Tables

**Table 1:** Calculated Diffusion coefficient, b-values, and series resistance values of α-Fe_2_O_3_, PVP-Fe, PEG-Fe, PVP/PEG-Fe electrodes.

Sample Code	Diffusion Coefficient (cm^2^/s) × 10^−8^		B-Value	ESR (Ω)
	Oxidation	Reduction		
**α-Fe_2_O_3_**	1.9	1.65	0.7	0.64
**PVP-Fe**	20.49	24.32	0.57	0.58
**PEG-Fe**	32.76	34.5	0.56	0.56
**PVP/PEG-Fe**	37.68	43.4	0.54	0.33

**Table 2:** Comparison of calculated areal capacitance, specific capacity and specific capacitance values of α-Fe_2_O_3_, PVP-Fe, PEG-Fe, PVP/PEG-Fe electrodes.

Sample Code	I (mA cm^−2^)	Areal Capacitance C_A_ (F cm^−2^)	Capacity (mAh cm^−2^)	Specific Capacitance C_S_ (F g^−1^)
**α-Fe_2_O_3_**	8	2.599	0.126	1299.5
	10	1.306	0.063	653
	20	1.078	0.052	539
	30	0.980	0.048	490
	40	0.588	0.029	294
	50	0.490	0.024	245
**PVP-Fe**	8	3.749	0.182	1874.5
	10	3.576	0.174	1788
	20	1.796	0.087	898
	30	1.224	0.060	612
	40	0.849	0.041	424.5
	50	0.735	0.036	367.5
**PEG-Fe**	8	5.682	0.276	2841
	10	4.408	0.214	2204
	20	4.057	0.193	2028
	30	3.429	0.167	1714.5
	40	2.743	0.133	1371.5
	50	1.633	0.079	816.5
**PVP/PEG-Fe**	8	9.143	0.651	4571.5
	10	8.980	0.437	4490
	20	8.088	0.406	4044
	30	7.116	0.369	3558
	40	6.531	0.317	3265.5
	50	4.898	0.238	2449

**Table 3:** Calculated areal capacitance, specific capacity, energy density, and power density values of PVP/PEG-Fe asymmetric pouch-type supercapacitor device.

Sample Code	I (mA)	CA (F cm^−2^)	C (mAh cm^−2^)	ED (mWh cm^−2^)	PD (mW cm^−2^)
**PVP/PEG-Fe device**	10	0.260	0.054	0.081	1.24
	20	0.103	0.021	0.032	1.29
	30	0.049	0.010	0.015	1.39
	40	0.021	0.004	0.007	1.20

## Data Availability

The data presented in this study are available on request from the corresponding authors.
